# PhyloFinder: An intelligent search engine for phylogenetic tree databases

**DOI:** 10.1186/1471-2148-8-90

**Published:** 2008-03-21

**Authors:** Duhong Chen, J Gordon Burleigh, Mukul S Bansal, David Fernández-Baca

**Affiliations:** 1 Department of Computer Science, Iowa State University, Ames, IA 50011, USA; 2 NESCent, Durham, NC 27705, USA

## Abstract

**Background:**

Bioinformatic tools are needed to store and access the rapidly growing phylogenetic data. These tools should enable users to identify existing phylogenetic trees containing a specified taxon or set of taxa and to compare a specified phylogenetic hypothesis to existing phylogenetic trees.

**Results:**

PhyloFinder is an intelligent search engine for phylogenetic databases that we have implemented using trees from TreeBASE. It enables taxonomic queries, in which it identifies trees in the database containing the exact name of the query taxon and/or any synonymous taxon names, and it provides spelling suggestions for the query when there is no match. Additionally, PhyloFinder can identify trees containing descendants or direct ancestors of the query taxon. PhyloFinder also performs phylogenetic queries, in which it identifies trees that contain the query tree or topologies that are similar to the query tree.

**Conclusion:**

PhyloFinder can enhance the utility of any tree database by providing tools for both taxonomic and phylogenetic queries as well as visualization tools that highlight the query results and provide links to NCBI and TBMap. An implementation of PhyloFinder using trees from TreeBASE is available from the web client application found in the availability and requirements section.

## Background

The rapidly expanding wealth of phylogenetic information from across the tree of life offers unprecedented opportunities for large-scale evolutionary studies and for examining an array of biological questions in a phylogenetic context [1]. However, much of the published phylogenetic data is not easily accessible. Therefore, the storage and efficient retrieval of phylogenetic data are important challenges for bioinformatics [1-5]. TreeBASE is the largest relational database of published phylogenetic information. It stores more than 4,400 trees that contain over 75,000 taxa, the data matrices used to infer the trees, and additional meta-data, such as bibliographic information and details of the phylogenetic analyses [6,7]. Though TreeBASE is a valuable repository for phylogenetic data, it is often difficult to identify and access relevant phylogenetic data from within TreeBASE. In this paper, we present PhyloFinder, a new phylogenetic tree search engine that greatly expands upon the current search features in TreeBASE and thus can enhance the utility of TreeBASE, or any phylogenetic database.

To utilize the existing phylogenetic data effiectively, we need tools that can quickly identify phylogenetic trees containing a specified taxon or set of taxa and that can compare a specified phylogenetic hypothesis to existing phylogenetic trees. The complexity of taxonomy presents a first major challenge for identifying and accessing phylogenetic data [3,4,6,7]. Taxonomic names used in stored phylogenetic trees often are based on various inconsistent taxonomies [6]. Furthermore, taxonomic classifications and names frequently change, and these changes may not be reflected in database trees. Consequently, repositories such as TreeBASE contain many species that are represented by multiple equivalent names. Taxonomic queries are further complicated by misspellings or unique subspecies designations in stored trees, both of which are common in TreeBASE [6]. Many of these taxonomic issues have been addressed by TBMap, a database that maps names of taxa found in TreeBASE to other taxonomic databases and clusters equivalent taxonomic names [6]. However, TBMap is not incorporated in TreeBASE or in any other phylogenetic search engines.

The hierarchical nature of taxonomic classifications presents further challenges for accessing phylogenetic data. The leaves in stored phylogenetic trees may represent different taxonomic levels, such as families, genera, species, or subspecies. It should be possible for a tree database query to identify trees containing not only the specific taxon name used in the query, but also trees containing descendants or ancestors of the query taxon [3,4]. For example, a query using the plant family name "Pinaceae" ideally would identify not only trees that contain the exact name "Pinaceae" but also trees containing Pinaceae genera such as *"Pinus" *or *"Abies" *or species such as *"Pinus thunbergii" *or *"Abies alba"*. It also would be useful to identify trees containing direct ancestors (the internal nodes on the path from the root of a taxonomy tree to the query taxon) of the query taxon. Thus, a query on the species name *"Pinus thunbergii" *would identify trees that contain the genus name *"Pinus" *or the family name "Pinaceae" as leaves. Currently, TreeBASE does not directly utilize information from taxonomic classifications to allow the user to find trees containing ancestors or descendants of the query taxon [3,4]. Instead, the user can find all the taxa matching a partial name taxon query. For example, querying "Pinus@" or even "Pinu@" in TreeBASE will identify all trees containing *"Pinus" *in their species name. However, querying using "Pinaceae@" will not identify trees with *"Pinus" *or *"Abies" *species, because they do not contain "Pinaceae" in the species name. Alternately, the user can identify trees with related taxa through "tree surfing", in which the user identifies neighboring trees (trees with shared taxa) of a specified tree(s). Tree surfing can be time consuming, and it is difficult if not impossible for the user to determine if s/he has found all the trees containing the relevant taxa.

Another important feature of an effective phylogenetic search engine is the ability to make phylogenetic queries, in which the user can assess a specified tree by comparing it to the trees in the database [3,5]. Tree mining queries must first be able to identify all trees that contain or agree with a query tree, or the trees in the database in which the query tree is embedded [3,4] (throughout this paper, the term 'mining' is used in the sense of searching). Additionally, since there is often much disagreement among trees, it is very useful to be able to identify all the trees that are similar, but not necessarily identical, to a query tree. Some tree mining features are implemented with TreeBASE [8-10].

In this paper, we introduce PhyloFinder, a search engine for phylogenetic databases that enhances the ability to search a tree database. PhyloFinder uses TBMap [6] to address the problem of taxonomic inconsistency, thereby expanding the power of taxonomic queries by recognizing synonymous taxon names. It also offers alternate spelling suggestions for taxonomic queries that do not find a match in the tree database. PhyloFinder further increases the querying power by using the hierarchical structure of the NCBI taxonomy [11] to search for trees containing descendants or ancestors of a query taxon, and it also enables a wide range of tree mining queries. PhyloFinder has a tree visualization tool that displays the query results, highlighting relevant taxa and branches, and provides hyperlinks to the NCBI taxonomy and TBMap websites. The implementation of PhyloFinder uses simple but powerful information retrieval techniques. These include the use of an inverted index that maps taxa to trees, which allows the system to filter out many trees that are irrelevant to a given query, and a representation of trees that allows fast least common ancestor queries directly on the database. We have tested PhyloFinder using trees from TreeBASE [12,13]. However, PhyloFinder can, in principle, be used with any phylogenetic database.

## Implementation

Before describing the implementation of PhyloFinder, we outline the features that it supports.

### Taxonomic Queries and Phylogenetic queries

PhyloFinder's web-based interface allows the user to make *taxonomic *and *phylogenetic *queries. Taxonomic queries involve a single taxon or set of taxa. Phylogenetic queries take as input a phylogenetic tree and attempt to locate trees in the database that match it in some specified way.

#### Taxonomic Queries

PhyloFinder supports three different types of taxonomic queries: *contains*, *related*, and *pathlength*.

**Figure 1:**
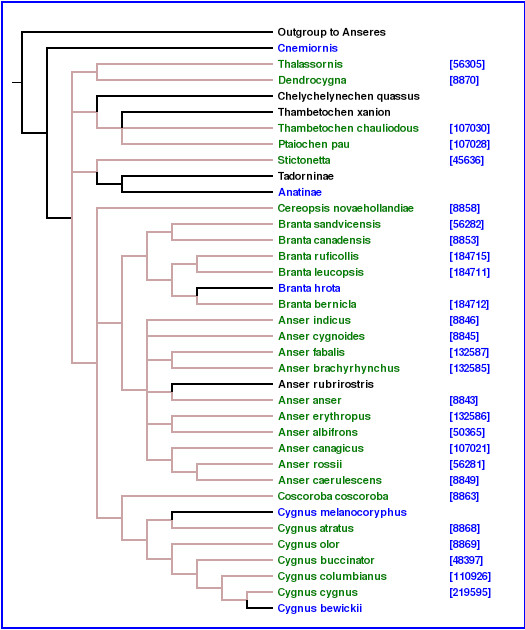
**Query result visualization**. One of the trees returned by querying for "birds" with the *related *command. The version of TreeBASE used by PhyloFinder contains around 88 such trees. Taxa in dark green are bird species that are found in the NCBI taxonomy database [11]. The blue numbers are the NCBI taxon ID numbers, and indicate hyperlinks to the NCBI taxonomy web site. The taxon names displayed in color (other than black) indicate hyperlinks to TBMap.

1. **Contains: **The input for this query is a set of taxon names, given as a comma-separated list. The output is a list of the tree IDs of all trees from the database that, depending on the user's choice, contain *all *or *any *of the taxon names in the set. Note: in our implementation of PhyloFinder using TreeBASE trees, the output is a list of the TreeBASE tree IDs and corresponding study IDs.

2. **Related: **The input for this query is a taxon name. The search engine finds all trees in the database involving any taxon that, depending on the user's choice, is a descendant or a direct ancestor of the query taxon in the NCBI taxonomy tree. For example, if the query taxon is "birds", and the user chooses the descendant option, the *related *command will identify all the trees in the database that contain any bird taxa [4] (Figure 1).

3. **Pathlength: **The input for this query is a pair of taxon names. The output is a list of the tree IDs of all trees containing the two species, along with the distance (path length) between the two taxa in each tree. Note: in our implementation of PhyloFinder using TreeBASE trees, the output is a list of the TreeBASE tree IDs, the corresponding study IDs, and the distance (path length) between the two taxa in each tree.

#### Phylogenetic Queries

PhyloFinder supports two different types of phylogenetic queries: *tree mining *and *tree similarity *search. To describe these commands, we need some definitions.

#### Definitions

**Figure 2:**
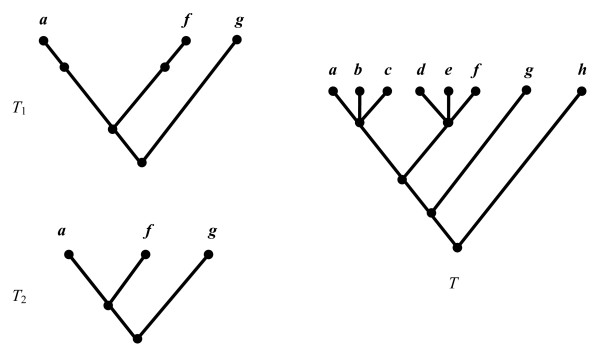
***T*(*A*) and *T|A***. For *A *= {*a, f, g*}, *T*_1 _is the tree *T*(*A*) and *T*_2 _is the tree *T*|*A*.

Let *T *be a phylogenetic tree, and *A *be a subset of the leaves of *T*. Following standard terminology [14], we write *T*(*A*) to denote the minimal subtree of *T *that contains the leaves in *A*, and *T*|*A *to denote the tree obtained from *T*(*A*) by suppressing all internal nodes that have only one child. An example is shown in Figure 2.

**Figure 3:**
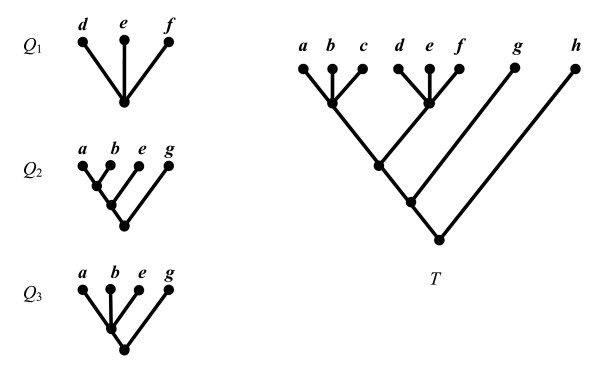
**Tree mining: pruned, embedded, and refined**. In this example, the query tree *Q*_1 _is a pruned subtree and an embedded subtree of tree *T*; *Q*_1 _is also (trivially) refined within *T*. The query tree *Q*_2 _is an embedded subtree of *T *and also (trivially) refined in *T*; however, it is not a pruned subtree of *T*. The query tree *Q*_3 _is refined by *T *but is neither an embedded nor a pruned subtree of tree *T*.

Let *Q *be a query tree and *T *be a candidate tree (that is, a tree from the database). Let *A *denote the set of leaves of *Q*. Tree *Q *is a *pruned subtree *of *T *if and only if either (i) *T *= *Q*, or (ii) there exists an edge in *T *which, when pruned, produces *Q *as the cut-out (i.e. pruned) subtree. This is illustrated in Figure 3. Tree *Q *is an *embedded subtree *of *T *if and only if it is identical to *T*|*A *(see Figure 3). Informally, this means that *Q *shows the same evolutionary relationships implied by *T*. Note that if *Q *is a pruned subtree of *T*, it must also be an embedded subtree, but the converse is not true (Figure 3). If *Q *is an embedded subtree of *T*, the tree *T*(*A*) is called the *embedding *of *Q *in *T*. We say that tree *Q *is *refined *by tree *T *(or that *T *refines *Q*) if the set of clusters of *Q *is a subset of the set of clusters of *T*|*A*; i.e., *T*|*A *is a refinement of *Q*. Note that if *Q *is an embedded subtree of *T*, it must also be refined by *T*, but not vice versa (Figure 3).

A *similarity measure *is a function that, given a *query tree *and a *candidate tree *returns a percentage score between 0 and 100%. This percentage is called a *similarity score *and it reflects how similar the query tree is to the candidate tree. PhyloFinder uses two similarity measures, one based on the Robinson-Foulds (RF) distance [15] and the other based on least common ancestors (LCAs). If the LCA-based score is 100%, the query tree is refined by the candidate tree, while if the RF similarity score is 100%, the query tree can be embedded in the candidate tree. These measures are described in the Appendix.

#### Commands

1. **Tree mining: **The input is a query tree *Q *in Newick format. The output is a list of tree IDs (TreeBASE tree IDs and study IDs in our current implementation) of all trees that exhibit the query tree *Q *in some way. There are three options for this command, which are listed below:

• Pruned: The output is a set of trees that contain *Q *as a pruned subtree.

• Embedded: The output is a set of trees that have *Q *as an embedded subtree. For the same query tree *Q*, the result of embedded subtree mining will be a superset of the output returned by the pruned subtree mining command.

• Refined: The output is all trees that refine *Q*. The result will be a superset of the output set returned by the embedded subtree mining command on the same query tree *Q*.

2. **Similarity: **The input is a tree in Newick format. The output is a list of the IDs of all trees that share at least three taxa with the query tree, ranked according to their similarity scores. Two options are provided, depending on whether the similarity score is computed with the RF-based or the LCA-based measure. (We require at least three shared taxa, because fewer than three taxa provide no topological information for rooted trees.)

### System Architecture

**Figure 4:**
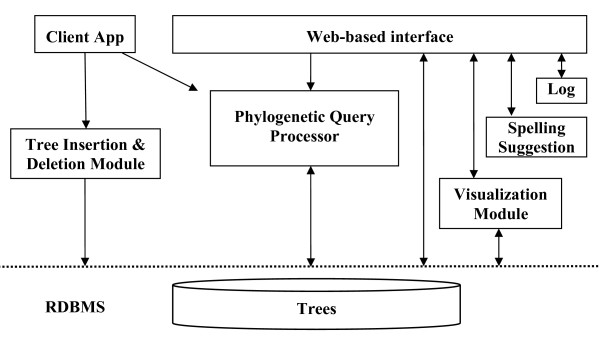
PhyloFinder's system architecture.

**Figure 5:**
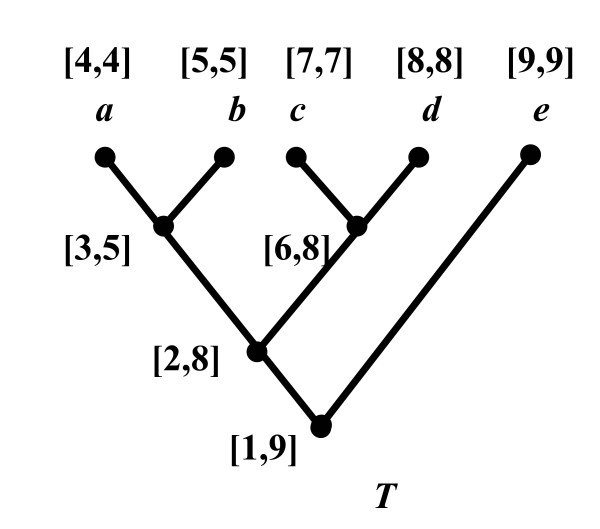
A slight modification of nested-set representation of a rooted tree.

Figure 4 shows the system architecture of PhyloFinder. The search engine is built on top of MySQL, an open-source relational database management system (RDBMS) [16]. PhyloFinder stores the phylogenetic trees, which in our test implementation are from TreeBASE [12,13], and the NCBI taxonomy tree [11] in MySQL using a slight modification [17] of *nested-set representation *[18,19]. Under this scheme, each node *x *of a given tree is represented by an interval [*N*_*x*_, *R*_*x*_], where *N*_*x*_, called the NodeID of *x*, is an integer defined by a preorder walk of the tree [20], and *R*_*x *_is the largest NodeID of a descendant of *x *(see Figure 5). Thus, node *y *is an ancestor of node *x *if and only if the interval [*N*_*y*_, *R*_*y*_] contains the interval [*N*_*x*_*, R*_*x*_], and the LCA of a set of nodes *x*_1_,...,*x*_*k *_is the common ancestor *y *with largest NodeID. With this representation, LCA queries can be implemented as SQL queries directly on the relational database. More efficient algorithms are available to solve the LCA problem [21]. However, our approach is directly supported by the database and it is fast in practice. (Alternately, we could have stored trees as lists of edges [5]; however, phylogenetic queries would have required recursive SQL extensions, which are not supported by many RDBMSs.)

The RDBMS also stores the NCBI taxon names. In order to automatically translate between synonymous names for the same species, the RDBMS stores a collection of *taxon clusters*, where each cluster contains a set of synonymous taxa. Taxon clusters are generated using TBMap [6] and the NCBI taxonomy database.

**Figure 6:**
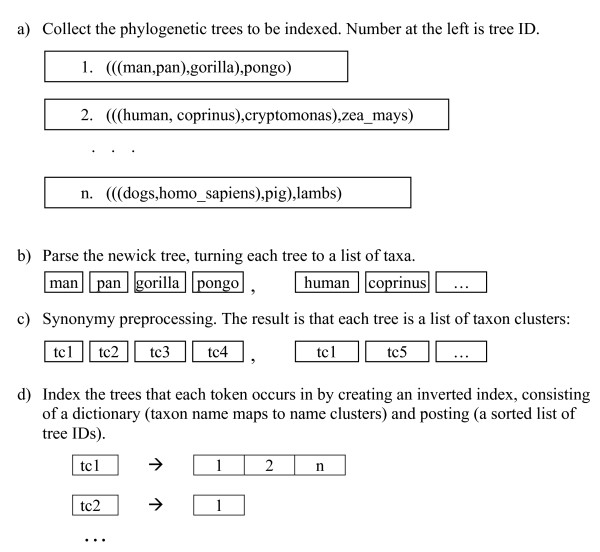
Construction of the inverted Index.

PhyloFinder uses an *inverted index *to achieve fast querying. In text retrieval and web mining, such indices are used as mappings from words to sets of documents that contain them [22]. PhyloFinder's inverted index treats taxon clusters as words and phylogenetic trees as documents (Figure 6).

The *phylogenetic query processor *is the kernel of the search engine. It provides functions to parse user commands, to perform taxonomic and phylogenetic queries, and to coordinate other modules. It relies heavily on the inverted index. The query processor uses the NCBI tree as a classification guide for species in queries such as the *related *command.

The *spelling suggestion module *checks spellings in the query and provides suggestions based on taxon names in TreeBASE or in the NCBI taxonomy database. This is implemented using the GNU Aspell [23] c library, with some modifications in order to handle special alphabet characters (e.g., '-', '&', '.') and compound words in taxon names.

The *visualization module *creates HTML files with image map, which show phylogenetic trees in a dendrogram format, highlight the embedded query tree or species, and provide link-outs for species in NCBI taxonomy database. While there are many available tree visualization tools [24-26], we could not find one that could easily be adapted to highlight query results and provide outlinks. Therefore, we developed a new tree visualization tool for PhyloFinder.

The *client application *is an administration tool that provides an interface to handle tree insertions and deletions (via the *insertion and deletion module*). It also provides commands for updating the inverted index and performing low-level queries (SQL commands or specified query commands with unformatted results) for testing and debugging.

The *web-based interface *is an interactive web application that uses the AJAX technique. It is written using the Google Web Toolkit (GWT) [27] and the GWT Window Manager (GWM) [28].

The system maintains a *log*, where it records user queries and timestamps. The statistics can be used to analyze user needs and help optimize the search engine performance.

### Taxonomic name consistency

**Figure 7:**
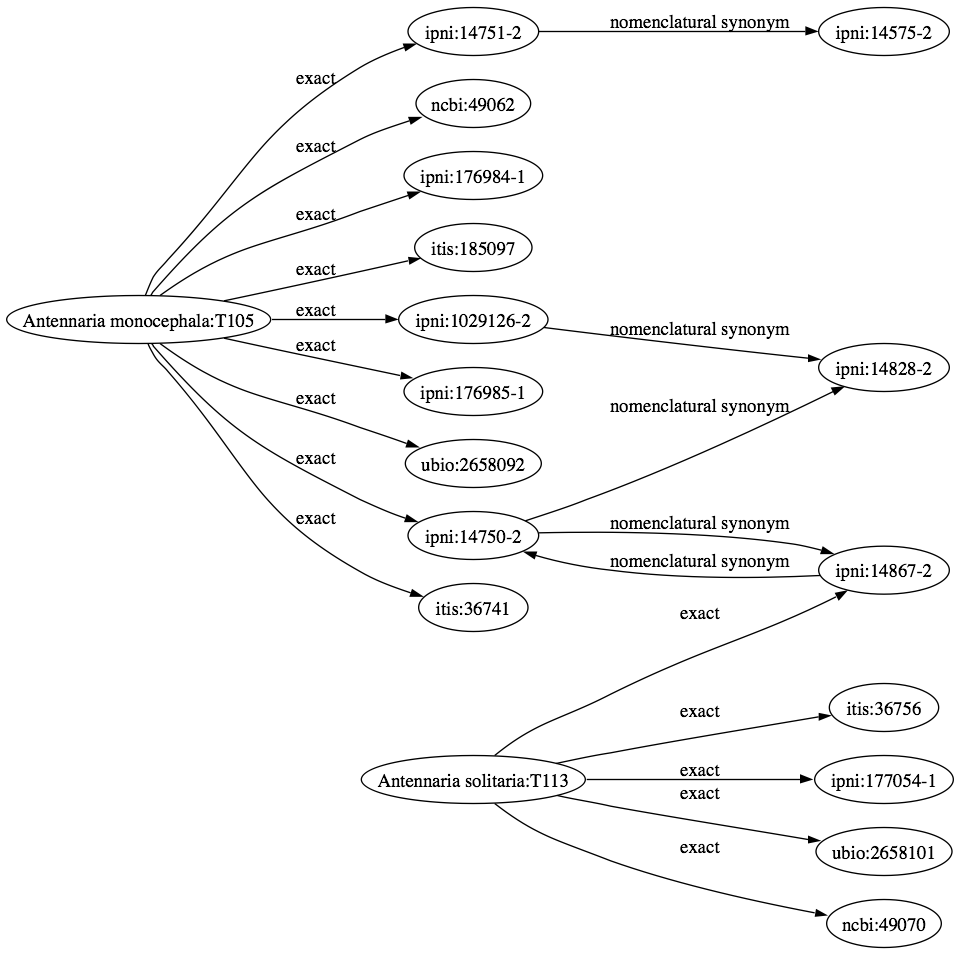
**Synonym conflict in IPNI and NCBI**. Name cluster containing "*Antennaria solitaria*" and "*Antennaria monocephala*" produced by TBMap [8]. *Anternnaria solitaria *and *Antennaria monocephala *are synonyms in IPNI, but are treated as distinct taxa by NCBI. In such cases, PhyloFinder uses the NCBI classification.

As mentioned above, the search engine relies on taxon clusters provided by TBMap [6] to identify synonymous taxonomic names. Ideally, TBMap and PhyloFinder should include the same set of taxon names as TreeBASE. However, TBMap is based on a 2004 snapshot of TreeBASE [6]. Thus, some new taxon names in TreeBASE are not included in TBMap. Whenever PhyloFinder encounters an inconsistency, it ignores the classification provided by TBMap, and relies instead on the NCBI taxonomy. We do this because the *related *query is based on the NCBI taxonomy, and using the NCBI taxonomy maximizes the utility of this feature. Another complication is that TBMap uses four external taxonomic databases – ITIS [29], IPNI [30], uBIO [31] and NCBI [11] – among which there are conflicts. For example, "*Antennaria solitaria*" and "*Antennaria monocephala*" are treated as synonyms in IPNI but not in NCBI (see Figure 7). In such cases, PhyloFinder again uses the NCBI classification.

### Query processing

**Figure 8:**
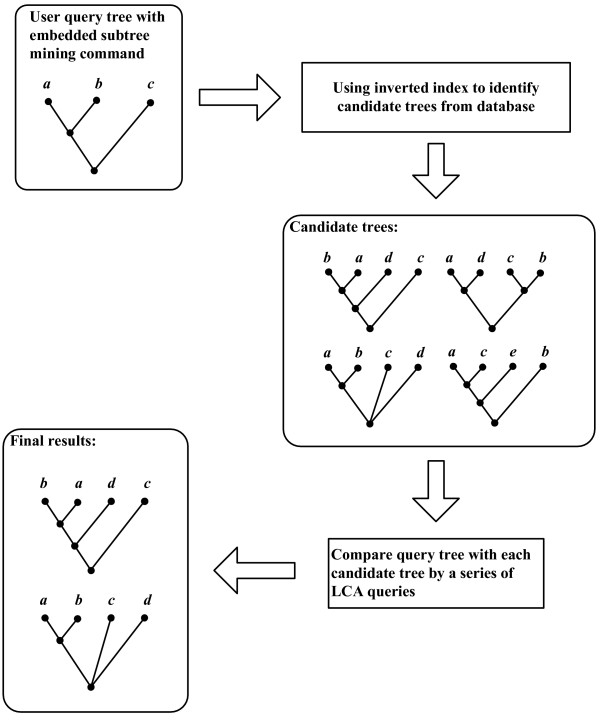
Processing queries through the search engine.

When a user submits a query from a web browser, the web CGI program parses the user query into query command (e.g., *contains*) and query contents (e.g., the taxa in the list). The results for some queries (such as obtaining the NCBI taxon ID for a given taxon name) can be retrieved directly from the database. Other queries (such as the *tree mining *command) go through the query processor, which first identifies candidate trees using the inverted index and then performs further computations to get the final results (see Figure 8). Identifying the candidate trees using the inverted index allows us to filter a large fraction of the database trees from further consideration, significantly accelerating the query processing.

**Figure 9:**
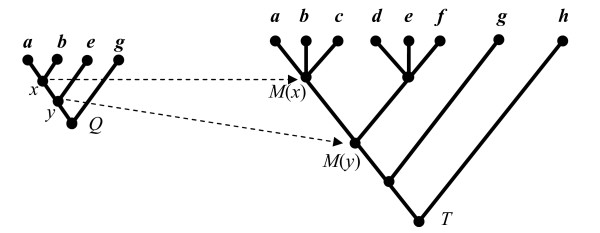
Tree mining using LCA mappings.

Phylogenetic queries (other than RF-based similarity search) are implemented by using LCA queries to compare ancestor-descendant relationships in the query tree and in the database tree. Figure 9 shows a simple example where the goal is to determine if query tree *Q *can be embedded in database tree *T*. The two internal nodes *x *and *y *in *Q *map to nodes *M*(*x*) and *M*(*y*) in *T *in the sense that the LCAs of the descendants of *x *and *y *are *M*(*x*) and *M*(*y*), respectively. *Q *can be embedded in *T *since *M*(*x*) and *M*(*y*) have the same ancestor-descendant relationship in *T *as *x *and *y *have in *Q*. The use of nested-set representation for trees in the RDBMS allows LCA queries to be directly computed by the RDBMS.

Hierarchical queries (e.g., *related*), which require a classification (or ontology) guide [3], are addressed by Boolean operations on the inverted index. For example, when a user query asks for trees that contain "birds" using the *related/descendant *command, the search engine first looks for all bird species in the stored phylogenetic trees using the NCBI taxonomy tree to identify all bird species in the trees. It then retrieves the tree ID lists corresponding to each bird species and returns their union (equivalent to Boolean "OR").

### Initial setup

While not fully automated, PhyloFinder's setup is reasonably straightforward and can, in principle, be used on any given data set of phylogenetic trees. The first step in the setup is to assemble all the phylogenetic trees in Newick format together in a file. A program then reads this file and converts the trees into the nested set representation used by PhyloFinder. The output of this program consists of several MySQL tables. A second program then reads in these tables, and uses data from additional sources, like TBMap and NCBI, to create some additional MySQL tables which help to improve query processing. All these MySQL tables must then be loaded into the MySQL database. Once this is done, the main program can be run. The main program first reads some tables from the MySQL database in order to create the inverted index, and then waits for commands from the client application. Once the server is set up, the system is ready for use.

In practice, we have found that setting up the system for TreeBASE data is more complicated. This is because TreeBASE data does not always conform to the Newick standard, and some of the trees contain errors. In addition, several of the trees contain special international characters. Dealing with this effectively requires some manual work and it makes it difficult to automatically update the local copy of TreeBASE.

## Results

**Figure 10:**
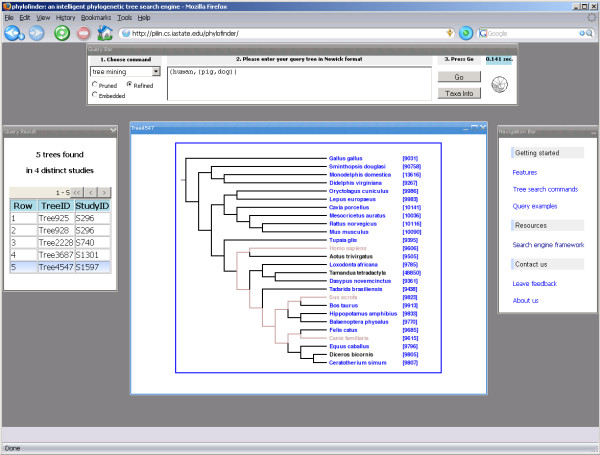
Screenshot of PhyloFinder's web interface.

We tested PhyloFinder using the trees from TreeBASE [12]. PhyloFinder's interactive web interface is shown in Figure 10. At the top center of the main window is a query panel that contains widgets for choosing commands and entering queries. At the right is a navigation bar that includes help links and links to information on the PhyloFinder framework. A result panel is displayed when the search engine finds results matching a query. Trees are displayed graphically when the user clicks the records in the result panel. Phylogenetic query results are visualized by highlighting the set *C *of taxa that the result tree and the query tree have in common, as well as edges in the result tree that connect *C*. If a taxon in the result tree is in TBMap, its name is hyperlinked to TBMap, and if the taxon is in the NCBI taxonomy database, an NCBI taxon ID number with a hyperlink to the NCBI taxonomy browser is appended to the taxon name.

Figures 1 and 10, illustrate various features of PhyloFinder. Note the use of color by the visualization module. Embedded query trees and taxon names are highlighted using various colors. Brown indicates an exact match (*i.e*. same taxon name or NCBI taxon ID). For the *related *command, ancestors of the query taxon are highlighted in orange, and descendants are highlighted in green.

**Figure 11:**
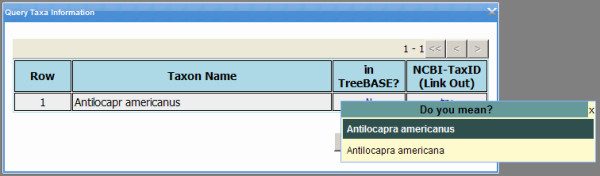
**Spelling suggestions**. Spelling suggestions for the query taxon "*Antilocapr americanus*".

The system provides spelling suggestions – all from TreeBASE and NCBI – for misspelled taxon names. For example, Figure 11 shows the spelling suggestions offered by PhyloFinder for the query taxon "*Antilocapr americanus*", which is neither in any of the TreeBASE trees nor in the NCBI database. When a user chooses a name from the suggestion list, PhyloFinder automatically updates the user query and the NCBI taxonomy out-link.

PhyloFinder effectively uses the NCBI taxonomy and the TBMap database to translate between different names for the same species (see Figure 10). For example, if one queries for trees that are similar to "(human,(pig, dog))", PhyloFinder will look for trees that contain scientific names for human, pig, and dog. TBMap allows us to find many synonyms that would be missed by using only the NCBI names.

There are 64,529 leaf taxa in the TreeBASE trees used by PhyloFinder. By itself, NCBI allows us to map 30,007 of these taxa to 29,669 distinct NCBI TaxonIDs, leaving 34,522 isolated taxa, each of which is in its own cluster, for a total of 64,191 clusters. In contrast, using TBMap we are able to map 36,864 leaf taxa in TreeBASE to NCBI taxa. The total number of taxon clusters is 52,198, of which 29,480 are mapped to NCBI taxa.

PhyloFinder uses the NCBI classification among species in user queries. Figure 1 shows an example of a taxon query looking for trees that contain species in "birds". The descendants of "birds" are highlighted in dark green in the displayed tree.

For the same query term, PhyloFinder normally returns more trees than TreeBASE even though PhyloFinder uses data from an older version of TreeBASE (containing fewer trees). For example, for a query on "angiosperms", TreeBASE returns 8 studies with 17 trees while PhyloFinder retrieves 543 studies and 1,550 trees using the *related *command; When querying on "Fungi", PhyloFinder returns 487 studies with 1,054 trees, as compared to the 6 studies with 15 trees that are retrieved using TreeBASE searching tools. This remarkable improvement over TreeBASE is achieved due to a combination of the classification guide provided by the NCBI taxonomy tree, and taxon cluster information from TBMap.

The time required to process a query depends on network speed, server load, client computer performance, among other factors. In most of our test queries, the results were received within a matter of seconds. In particular, elapsed times and CPU times for a taxonomic query on "angiosperms" using the *related *command were 0.266s and 0.076s respectively; and for "Fungi" the corresponding times were 0.172s and 0.06s respectively.

## Discussion

PhyloFinder enhances the utility of TreeBASE by making it easier to assess and obtain the phylogenetic data contained within it. PhyloFinder adds several new taxonomic querying capabilities to TreeBASE, including spelling suggestions, searches for synonymous taxonomic names, and the ability to identify trees with ancestors or descendants of the query taxon and to identify path lengths between taxa in the database trees. PhyloFinder also expands the power of phylogenetic queries, offering more precise options for identifying different types of subtrees and more metrics for identifying similar trees than TreeBASE. Additionally, PhyloFinder provides a tree visualization tool that highlights query taxa in an informative manner and gives useful outlinks to GenBank and TBMap. Furthermore, PhyloFinder provides nearly immediate results for most queries. Still, PhyloFinder is a search engine, not a database, and is not meant to be a substitute for tree repositories such as TreeBASE.

PhyloFinder is not limited to TreeBASE and can be incorporated into any phylogenetic tree database. For example, we have incorporated PhyloFinder into the PhyLoTA browser for gene trees (Sanderson M, Boss D, Chen D, Cranston KA, Wehe A: The PhyLoTA browser: processing GenBank for molecular phylogenetics research, submitted) [32]. Future development of PhyloFinder will include a desktop version in which users can input their own sets of phylogenetic trees. A number of other extensions to the search engine are also under development. These include handling unrooted trees, providing an interface for retrieving trees in Newick format and more options for drawing trees, displaying details of the phylogenetic study in the query results, and ranking trees returned by the *embedded subtree mining *command (e.g., the rank of a tree *T *could be the number of edge contractions of *T *that are required to get the query tree). The search engine can also be linked to tools for building supertrees, which will allow users to assemble large phylogenies by combining phylogenetic trees with incomplete taxon overlap.

The usefulness of any phylogenetic search engine is limited by the amount of phylogenetic information it can search. Although TreeBASE is the largest relational database of published phylogenetic trees, few journals require that trees be submitted to TreeBASE, and thus it contains only a small percentage of published phylogenetic trees. We hope that the development of more effective methods to access and utilize phylogenetic data will further motivate efforts to collect and store phylogenetic information.

## Conclusion

While there has been great progress in understanding organismal relationships across the tree of life, this phylogenetic data is often not easily accessible to scientists. PhyloFinder enhances the utility of phylogenetic databases, by enabling scientists to identify and assess available phylogenetic data. The taxonomic search tools in PhyloFinder allow researchers to identify all trees containing taxa of interest without knowing the names of all taxa in the available trees, possible synonymous taxon names, or even the correct spelling of a taxon name. The phylogenetic search tools allow one to evaluate phylogenetic hypotheses by comparing it to existing phylogenies and identifying trees that agree with or are similar to the query tree. We have tested the utility of PhyloFinder using trees from TreeBASE, the largest relational database of published phylogenetic information, but the search engine can be used with any other tree database.

## Availability and requirements

A PhyloFinder server for TreeBASE trees is set up at Iowa State University. The web client application is available at http://pilin.cs.iastate.edu/phylofinder/. All the features and functions described in this manuscript are freely accessible from the web site. A detailed diagram of the database scheme is available at: http://pilin.cs.iastate.edu/phylofinder/phylofinder-schema.pdf

### System requirements

PhyloFinder's web application has been tested with Microsoft Internet Explorer version 6.0 and above, and with Mozilla Firefox version 1.5 and above. Some minor issues with the web interface may occur using Apple Safari.

## Authors' contributions

DC was the primary designer and implementer of PhyloFinder, and wrote major parts of the paper. JGB was involved in the system design and the writing of the manuscript. MSB contributed in the initial stages of the system design, conceived the LCA-based similarity measure used by the system, helped with the design and implementation of phylogenetic queries, and contributed to the writing of the paper. DFB contributed to the system design and the writing of the paper. All authors read and approved the final manuscript.

## Appendix: Similarity measures

Here we describe the two similarity measures used by our system. The first of these is based on the well-known Robinson-Foulds distance. The other uses the notion of a *Least Common Ancestor *(LCA). The LCA of a set of nodes in a tree is the most recent node in the tree whose descendants include all elements of the set.

In what follows, *Q *denotes the query tree and *T *denotes the candidate (database) tree. We write *n*(*T*) to denote the total number of taxa in tree *T*. *C *denotes the set of taxa that *T *and *Q *share in common and *n*(*C*) is the number of elements in *C*.

### Robinson-Foulds similarity

If *T *and *Q *have the same set of taxa, then the Robinson-Foulds distance between them is the number of clusters in *T *that do not appear in *Q *plus the number of clusters in *Q *that do not appear in *T*. In practice, one must take into account the fact that the taxon overlap *C *between *T *and *Q *may be only partial. Thus, we define the (Robinson-Foulds) similarity between *T *and *Q *as

$$similarity_{RF}(T,Q)=\{\begin{array}{cc} 0 & \begin{array}{cc} \text{if} & r=0 \end{array}\\ \frac{n(C)}{n(T)}\times \left(1-\frac{RF(T|C,Q|C}{r}\right)\times 100\% & \text{otherwise} \end{array}$$

where *RF*(*T*|*C*, *Q*|*C*) is the Robinson-Foulds distance between *T*|*C *and *Q*|*C *and *r *is the total number of non-trivial clusters in *T*|*C *and *Q*|*C*.

### LCA-based similarity

This measure is based on an LCA-based mapping from nodes in *Q *to nodes in *T*. The mapping assigns to each node *x *in *Q *a node *M*(*x*) in *T*. This is done as follows. Let *x *be the set of all leaf-descendants of *x *in *Q*. Then, *M*(*x*) is the LCA of set *x *in *T*. Node *x *in *Q *is said to be a *conflicting node *(with respect to *T*) if it has a sibling *y *such that *M*(*x*) is an ancestor of (or equal to) *M*(*y*) in the candidate tree *T*, *i.e*. *M*(*x*) and *M *(*y*) are the same node or two nodes that have an ancestor-descendant relationship in *T *but such that *x *and *y *are siblings in *Q*. Our search engine can find conflicting nodes rapidly because the storage mechanism it employs supports quick LCA calculation and fast determination of the ancestor/descendant relationship between any two nodes.

The number of conflicting nodes can vary between 0 and *n*-1, where *n *is the number of internal nodes in the query tree. If node *x *is conflicting, then *x *has a sibling *y *such that the leaf clusters defined by *x *and *y *do not induce disjoint clades in the candidate tree. Thus, the percentage of conflicting nodes is a measure of the level of agreement between the query tree *Q *with the candidate tree *T*.

To define a practical similarity measure, we must take into account the degree of overlap between the query tree *Q *and the candidate tree *T*. This is done as follows. Let *q *denote the number of internal nodes in *Q*|*C*, and *p *denote the number of conflicting nodes in *Q*|*C *with respect to tree *T*. Observe that the number of conflicting nodes is at most *q *- 1. Then, the similarity score of query tree *Q *to candidate tree *T *is given as follows.

$$similarity_{LCA}(Q,T)=\{\begin{array}{cc} \frac{n(C)}{n(Q)}\times 100\% & \begin{array}{cc} \text{if} & q=1 \end{array}\\ \frac{n(C)}{n(Q)}\times \left(1-\frac{p}{q-1}\right)\times 100\% & \text{otherwise} \end{array}$$

Here, the term $1-\frac{p}{q-1}$ captures the normalized value of the conflict in tree *Q*|*C *with respect to tree *T*.
